# Divergent evolution of arrested development in the dauer stage of *Caenorhabditis elegans *and the infective stage of *Heterodera glycines*

**DOI:** 10.1186/gb-2007-8-10-r211

**Published:** 2007-10-05

**Authors:** Axel A Elling, Makedonka Mitreva, Justin Recknor, Xiaowu Gai, John Martin, Thomas R Maier, Jeffrey P McDermott, Tarek Hewezi, David McK Bird, Eric L Davis, Richard S Hussey, Dan Nettleton, James P McCarter, Thomas J Baum

**Affiliations:** 1 Interdepartmental Genetics Program, Iowa State University, Ames, IA 50011, USA; 2 Department of Plant Pathology, Iowa State University, Ames, IA 50011, USA; 3 Current address: Department of Molecular, Cellular and Developmental Biology, Yale University, New Haven, CT 06520, USA; 4 Department of Genetics, Washington University School of Medicine, Genome Sequencing Center, St Louis, MO 63108, USA; 5 Department of Statistics, Iowa State University, Ames, IA 50011, USA; 6 LH Baker Center for Bioinformatics and Biological Statistics, Iowa State University, Ames, IA 50011, USA; 7 Current address: Center for Biomedical Informatics, The Children's Hospital of Philadelphia, Philadelphia, PA 19104, USA; 8 Current address: The University of Kansas Medical Center, Kansas City, KS 66160, USA; 9 Department of Plant Pathology, NC State University, Raleigh, NC 27695, USA; 10 Department of Plant Pathology, University of Georgia, Athens, GA 30602, USA; 11 Divergence Inc., North Warson Road, St Louis, MO 63141, USA

## Abstract

**Background:**

The soybean cyst nematode *Heterodera glycines* is the most important parasite in soybean production worldwide. A comprehensive analysis of large-scale gene expression changes throughout the development of plant-parasitic nematodes has been lacking to date.

**Results:**

We report an extensive genomic analysis of *H. glycines*, beginning with the generation of 20,100 expressed sequence tags (ESTs). In-depth analysis of these ESTs plus approximately 1,900 previously published sequences predicted 6,860 unique *H. glycines *genes and allowed a classification by function using InterProScan. Expression profiling of all 6,860 genes throughout the *H. glycines *life cycle was undertaken using the Affymetrix Soybean Genome Array GeneChip. Our data sets and results represent a comprehensive resource for molecular studies of *H. glycines*. Demonstrating the power of this resource, we were able to address whether arrested development in the *Caenorhabditis elegans *dauer larva and the *H. glycines *infective second-stage juvenile (J2) exhibits shared gene expression profiles. We determined that the gene expression profiles associated with the *C. elegans *dauer pathway are not uniformly conserved in *H. glycines *and that the expression profiles of genes for metabolic enzymes of *C. elegans *dauer larvae and *H. glycines *infective J2 are dissimilar.

**Conclusion:**

Our results indicate that hallmark gene expression patterns and metabolism features are not shared in the developmentally arrested life stages of *C. elegans *and *H. glycines*, suggesting that developmental arrest in these two nematode species has undergone more divergent evolution than previously thought and pointing to the need for detailed genomic analyses of individual parasite species.

## Abstract 2

The generation and analysis of over 20,000 ESTs allowed the identification and expression profiling of 6,860 predicted genes in the nematode *Heterodera glycines*. This revealed that gene expression patterns in the dauer stage of *Caenorhabditis elegans* are not conserved in *H. glycines*.

## Background

*Heterodera glycines*, the soybean cyst nematode, is the economically most important pathogen in soybean production and causes estimated annual yield losses of $800 million in the USA alone [1]. *H. glycines *completes its life cycle in about one month [2]. The first molt of the larvae takes place inside the eggs, and, after hatching, infective second-stage juveniles (J2) migrate through the soil and invade soybean roots to become parasitic J2. Once inside host roots, J2 move intracellulary through the root tissue to the central cylinder, where they initiate the formation of feeding sites (syncytia) and become sedentary. Only after feeding commences do nematodes molt and pass through two more juvenile stages (J3, J4) and, after a final molt, develop into adults. The enlarging body of the female, which remains sedentary for the remainder of the life cycle, breaks through the root cortex into the rhizosphere. Males regain motility and leave the root to fertilize females. After fertilization, females produce eggs, the majority of which are retained inside the uterus. Upon death of the adult female, its outer body layers harden and form a protective cyst (hence the name cyst nematodes) around the eggs until the environment is favorable again for a new generation of nematodes [2,3]. Even though eggs in their cysts are the primary dispersal stage of this nematode in an epidemiological sense, the J2 stage is mobile and, thus, comparable to the dispersal stage of *Caenorhabditis *spp.

In the past, numerous reports on cyst nematodes (*Heterodera *spp. and *Globodera *spp.) focused on selected genes, rather than taking a genomic approach, to elucidate nematode biology or the host-pathogen interactions between these nematodes and their host plants. Many of these studies dealt with so-called parasitism genes that are expressed in the dorsal and subventral esophageal glands during parasitic stages of cyst nematodes. The products of these genes are thought to be secreted into the host tissue to mediate successful plant parasitism [4-13]. However, a comprehensive genomic analysis beyond this limited group of genes has been lacking to date. To fill this gap, we generated 20,100 *H. glycines *expressed sequence tags (ESTs). Analyses of these ESTs plus approximately 1,900 sequences already in public databases produced a grouping into 6,860 unique genes. We assigned putative functions to these genes based upon sequence homology and established their expression profiles throughout the major life stages of *H. glycines*. Our data sets and results now represent a comprehensive resource for molecular studies of *H. glycines*.

Genomic analyses provide powerful tools to elucidate relationships between plant-parasitic nematodes and their hosts. Previous reports focused on the analysis of ESTs of plant-parasitic nematodes [14-17] or used differential display [18,19] and microarrays [20-22] to study gene expression changes in *Arabidopsis *and soybean in response to cyst nematode infection. Only recently, the advent of the Affymetrix Soybean Genome Array GeneChip enabled a parallel analysis of gene expression changes in both soybean and soybean cyst nematode during the early stages of infection [23]. The Affymetrix Soybean Genome Array GeneChip contains 37,500 probesets from soybean plants and additionally 15,800 probesets from the oomycete *Phythophthora sojae *and 7,530 probesets from the soybean cyst nematode *H. glycines*, two of the most important soybean pathogens. The *H. glycines *sequences used for the GeneChip have been generated in the study presented here.

The completion of the *C. elegans *genome sequence [24] was a milestone for biology at large, but it especially set the stage for comparisons to other (for example, parasitic) nematode species and has ushered in an era of comparative genomics in nematology [25-29]. One question of particular interest is whether the dauer larva, a facultative stage in the free-living species *C. elegans*, is homologous to the obligate dauer stage in parasitic nematodes. Dauer larvae were first described [30] as an adaptation to parasitism to overcome adverse environmental conditions and facilitate dispersal, but have been best studied in *C. elegans*. Genetic analysis has revealed the pathway controlling entry to and exit from the dauer stage [31]. This biochemical pathway, which is highly conserved across the animal kingdom, including humans [32], assesses and allocates energy resources to nematode development, ageing and fat deposition. The dauer pathway is primarily neuronally mediated, but presumably communicates with endocrine functions.

There is no strict definition of a 'dauer', but these larvae share the properties of being developmentally arrested, motile, non-feeding, non-ageing and hence long-lived [31,33,34]. Dauer stages have been well-documented for some plant-associated genera, including *Anguina *[35] and *Bursaphelenchus *[36], and it has been proposed that the infective stages of the sedentary endo-parasitic forms, including *H. glycines*, function as dauers [37]. In addition to the developmental attributes of the dauer, *H. glycines *J2 exhibit detergent resistance [38], intestinal morphology with sparse luminal microvilli [39] and numerous lipid storage vesicles characteristic of *C. elegans *dauers.

The dauer larva stage in *C. elegans *has distinct metabolic hallmarks [31]. Enzymes involved in the citrate cycle (with the exception of malate dehydrogenase) are less active in dauer larvae relative to adult *C. elegans*. Dauers show an increased level of phosphofructokinase activity and, therefore, glycolysis relative to adults [40]. The citrate cycle is less active than the glyoxylate cycle in dauer larvae compared to adults, consistent with the important role of lipids in energy storage in the dauer stage [41]. Also, heatshock protein 90 (Hsp90) is up-regulated fifteen-fold in dauer larvae relative to other stages [42], and superoxide dismutase and catalase activities show significant increases as well [43,44]. Although it is widely assumed that the dauer pathway *per se *is utilized to regulate dauer entry/exit in various animal-parasitic [45,46] and plant-parasitic species [26], little is known in these diverse species about the nature of the biochemistry that is regulated by the dauer pathways (that is, the effectors). Intriguingly, one experimental study in the human-parasitic nematode *Strongyloides stercoralis *[27] could not find clear evidence for a conserved dauer gene-expression signature, suggesting that the effectors of dauer biology might be diverged across nematode species.

However, a common feature among the dauer stage of *C. elegans *and the infective stage of parasitic nematode species seems to be the down-regulation of collagens, which make up a major portion of the nematode cuticle [27]. Collagens share a high degree of sequence identity due to numerous repeats, but they are not functionally redundant and often are developmentally regulated [47-50]. Previous EST studies found just three collagen transcripts in the infective stage of *Meloidogyne incognita *[14] and none in the infective stage of *S. stercoralis *[27]. In *C. elegans*, collagens could not be identified among dauer-specific transcripts [51].

Determining whether developmental arrest in *C. elegans *and parasitic nematodes like *H. glycines *is executed via the same mechanisms is a fundamental question of nematode biology. Of more than just academic interest, it may have important ramifications for potential control strategies that focus on the dauer pathway as a promising biochemical target to disrupt parasitic nematode life cycles. For example, it is very appealing to envision a strategy to induce dauer exit and concomitant resumption of ageing and development in the absence of a suitable host.

Here, we analyze and compare for the first time global gene-expression changes throughout all major life stages (eggs, infective J2, parasitic J2, J3, J4, virgin females) except adult males of a parasitic nematode and compare expression profiles to those of the model nematode *C. elegans*, with a particular focus on developmental arrest using EST and microarray data. Taken together, the sequence generation, sequence analyses and expression profiling work presented in this paper represent the most comprehensive and informative genomic resource available for the study of cyst nematode development and parasitism to date.

## Results

### EST generation and sequence analysis

Life stage-specific (eggs, infective J2, J3, J4, virgin females) cDNA libraries of *H. glycines*, the soybean cyst nematode, were generated to provide templates for EST sequencing, totaling 20,100 5' ESTs or almost 10 million nucleotides (GC content 48.9%). Sequences from all five developmental stages were represented in approximately equal proportions (Table 1). In addition to these stage-specific libraries, 1,858 *H. glycines *sequences previously deposited in GenBank were included in the dataset for this study, bringing the total number of sequences analyzed here to 21,958. This dataset was used by Affymetrix (Santa Clara, CA, USA) to form 6,860 unique contigs (average length 552 nucleotides, average size 3 ESTs), which then were represented by 7,530 probesets on the Affymetrix Soybean Genome Array GeneChip (gene discovery rate 31%; 6,860/21,958). Of the 6,860 unique contigs, 3,499 consisted of only one EST, so-called singletons (16% of all ESTs analyzed). On the other extreme, contig number HgAffx.13905.2 was formed by 599 ESTs. Furthermore, the 40 contigs that contained the largest number of ESTs represented 8.3% of all ESTs studied (Table 2).

**Table 1:** Properties of *H. glycines *cDNA libraries

*H. glycines *library	ESTs	Nucleotides (million)	Average length, standard deviation (nt)
Egg	3,636	2.06	568 ± 131
Infective J2	4,313	1.77	410 ± 135
J3	3,340	1.75	524 ± 144
J4	4,940	2.46	498 ± 147
Virgin female	3,871	1.93	498 ± 149

**Table 2:** The 40 most abundant *H. glycines *transcripts

Contig	EST	Contig length	E-value*	Identity (%)	Description
HgAffx.13905.2	599	846	4.4e-36	89.3	emb|CAB88203.1| Putative cuticular collagen [*Globodera pallida*]
HgAffx.18740.1	351	1,386^†^	3.5e-201	100	gb|AAN15196.1| Actin [*Globodera rostochiensis*]
HgAffx.13471.1	232	1,290^†^	4.3e-190	98.6	gb|AAO49799.1| Arginine kinase [*Heterodera glycines*]
HgAffx.7395.1	91	1,538	2.8e-202	100	gb|AAT70232.1| unc-87 [*H. glycines*]
HgAffx.3699.1	86	695^†^	1.4e-60	100	gb|AAO33474.1| Gland-specific protein g4g12 [*H. glycines*]
HgAffx.24400.1	81	756			Novel
HgAffx.22869.1	78	1,315	1.8e-177	82	sp|P49149| 60S ribosomal protein L3 [*Toxocara canis*]
HgAffx.13905.1	68	1,199	3.9e-25	43	emb|CAE70235.1| Hypothetical protein CBG16724 [*Caenorhabditis briggsae*]
HgAffx.11519.1	66	1,937	9.6e-24	75.3	ref|XP_453836.1| Unnamed protein product [*Kluyveromyces lactis*]
HgAffx.15767.1	64	1,242	3.0e-88	68.7	emb|CAC33829.1| Annexin 2 [*G. pallida*]
HgAffx.17330.1	61	486			Novel
HgAffx.13471.2	57	1,237	8.4e-128	82.3	gb|AAB38001.1| Hypothetical protein T01B11.4 [*Caenorhabditis elegans*]
HgAffx.20336.3	55	2,138	4.0e-66	45.1	dbj|BAB33421.1| Putative senescence-associated protein [*Pisum sativum*]
HgAffx.22036.1	54	1,087	2.8e-94	78.2	gb|AAF99870.1| Ribosomal small subunit protein 3 [*C. elegans*]
HgAffx.19294.1	53	672	4.2e-27	46.7	gb|AAK21484.1| Lipid binding protein 6 [*C. elegans*]
HgAffx.10986.1	51	1,311	4.5e-151	94.1	gb|AAC79129.1| Glyceraldehyde-3-phosphate-dehydrogenase [*G. rostochiensis*]
HgAffx.13471.3	48	2,514			Novel
HgAffx.20065.1	48	2,092^†^	0	95.6	gb|AAG47839.1| Heatshock protein 70 [*H. glycines*]
HgAffx.16311.1	47	2,368	2.3e-37	26.3	sp|Q94637| Vitellogenin 6 precursor [*Oscheius brevis*]
HgAffx.22005.5	44	615	2.0e-20	57.4	gb|AAL78212.1| Putative gland cell secretory protein Hgg-25 [*H. glycines*]
HgAffx.22952.1	44	565	2.9e-53	79	gb|AAT92172.1| Ribosomal protein S14 [*Ixodes pacificus*]
HgAffx.20012.1	44	486			Novel
HgAffx.16586.1	39	481			Novel
HgAffx.24042.1	38	474	3.1e-47	90.5	emb|CAA90434.1| Hypothetical protein C09H10.2 [*C. elegans*]
HgAffx.24357.1	37	479	4.1e-24	66.2	emb|CAE71709.1| Hypothetical protein CBG18686 [*C. briggsae*]
HgAffx.20747.1	37	1,145^†^	9.8e-88	72.4	gb|AAQ12016.1| Tropomyosin [*H. glycines*]
HgAffx.8887.1	36	788	5.9e-76	67.4	emb|CAE71139.1| Hypothetical protein CBG17994 [*C. briggsae*]
HgAffx.21332.1	36	2,325	0	91	gb|AAO14563.2| Heatshock protein 90 [*H. glycines*]
HgAffx.18233.1	35	913	5.5e-69	83.6	gb|AAL40718.1| Myosin regulatory light chain [*Meloidogyne incognita*]
HgAffx.23479.1	33	595	7.2e-39	73.1	gb|AAF08341.1| Peptidyl-prolyl cis-trans isomerase [*Brugia malayi*]
HgAffx.13457.1	33	783	4.6e-67	70.5	emb|CAE58579.1| Hypothetical protein CBG01745 [*C. briggsae*]
HgAffx.15145.1	33	2,162	4.5e-153	62.5	emb|CAA90444.1| Hypothetical protein F18H3.3a [*C. elegans*]
HgAffx.24295.1	33	1,043	4.7e-44	80.9	sp|P92504| Cytochrome c type-1 [*Ascaris suum*]
HgAffx.20336.1	32	1,225^†^	3.2e-157	92.7	gb|AAC48326.1| Beta-1,4-endoglucanase-2 precursor [*H. glycines*]
HgAffx.14833.1	31	1,440	3.0e-84	74	emb|CAA51679.1| Ubiquitin [*Lycopersicon esculentum*]
HgAffx.19292.1	30	749	7.9e-52	64.5	emb|CAE70207.1| Hypothetical protein CBG16683 [*C. briggsae*]
HgAffx.10017.1	30	846	1.0e-38	89.1	emb|CAB88203.1| Putative cuticular collagen [*G. pallida*]
HgAffx.19634.1	29	782	6.8e-61		emb|CAE64949.1| Hypothetical protein CBG09780 [*C. briggsae*]
HgAffx.24169.1	29	623			Novel
HgAffx.22005.1	28	765^†^	3.9e-114	90.7	gb|AAP30835.1| Putative gland protein G33E05 [*H. glycines*]

*1e-20 threshold. ^†^Full-length sequence.

**Figure 1:**
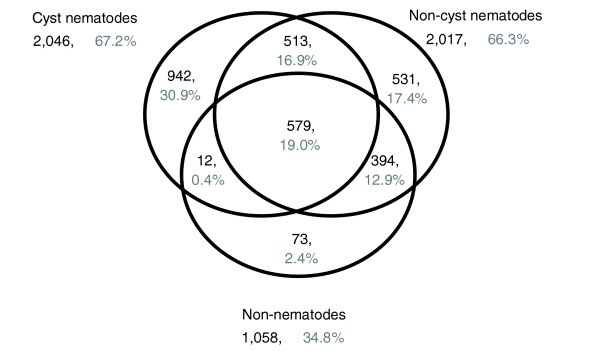
Venn diagram showing distribution of *H. glycines *BLAST hits by database. Forty-four percent of all 6,860 *H. glycines *contigs matched sequences in at least one of three databases at a threshold value of 1^e-20^: **(a) **All cyst nematodes without *H. glycines*. **(b) **All non-cyst nematodes. **(c) **All non-nematodes.

In order to determine sequence similarities of our contigs and in particular to identify genes that are conserved between different nematode species, we BLAST searched the 6,860 *H. glycines *contigs versus three databases (Figure 1). About half of the contigs (44%) matched sequences in at least one of these three databases at a threshold value of E = 1e^-20^. Examination of the BLAST match distribution revealed that 19% of the contigs that matched all three databases are most likely representing highly conserved genes involved in fundamental housekeeping processes in metazoans, while the 31% of contigs exclusively matching sequences in the cyst nematode database contained genes that likely are important for specific host adaptations of *Heterodera *spp.

When assessing BLAST hit identities, the cluster that contained the most ESTs (HgAffx.13905.2; 599 ESTs) belonged to a gene coding for a putative cuticular collagen. Identities of other highly represented contigs were actin, tropomyosin and myosin, as well as additional house-keeping genes like ribosomal components, ubiquitin, arginine kinase, synaptobrevin and heat shock proteins. Interestingly, four putative parasitism gene sequences from the esophageal gland cells were among the 40 contigs with the highest EST constituents: three of unknown function (AAO33474.1, AAL78212.1, AAP30835.1) [7,8] and a *β*-1,4-endoglucanase-2 precursor.

We further determined which *H. glycines *genes showed the highest degree of conservation when compared to *C. elegans*. BLASTX searches of all 6,860 soybean cyst nematode contigs against the Wormpep database revealed that 34.9% matched *C. elegans *entries at a threshold value of E = 1e^-20^. The products of the 25 most conserved genes were heat shock proteins, proteins related to transcription and translation (for example, elongation and splicing factors and RNA polymerase II) and structural proteins, including tubulin and actin, as well as enzymes, including guanylate cyclase (Additional data file 3).

### A survey and functional classification of developmentally regulated genes

**Figure 2:**
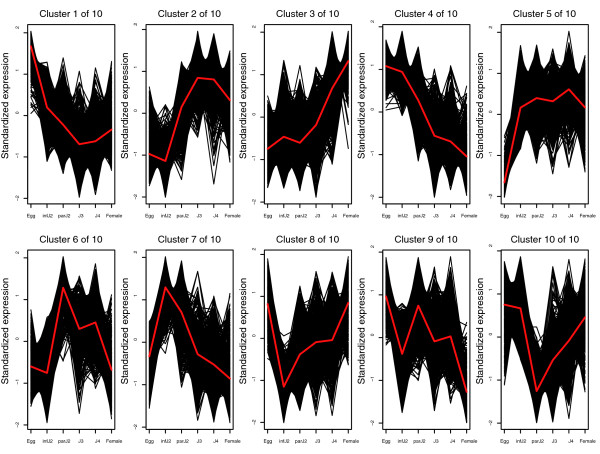
Differentially expressed *H. glycines *probesets. Temporal expression patterns of 6,695 *H. glycines *probesets that are differentially expressed (FDR 5%) when observed over the entire life cycle. Probesets were placed into ten clusters based on their temporal expression patterns. The average expression pattern of the probesets in each cluster is indicated by a red line. For visualization purposes, each probeset's estimated mean log-scale expression profile was standardized to have mean 0 and variance 2 prior to plotting. infJ2, infective J2; parJ2, parasitic J2.

In order to identify *H. glycines *genes that are developmentally regulated and to document their expression profiles, we designed a microarray experiment using three complete and independent biological replications (that is, three independent sample series representing three complete life cycles). We identified 6,695 probesets (Additional data file 4) as described in Materials and methods that were differentially expressed with a false discovery rate (FDR) of 5% when observed over the entire life cycle of *H. glycines*. This group of probesets equals 89% of all *H. glycines *probesets on the microarray. In other words, the vast majority of *H. glycines *genes represented on the GeneChip significantly changed expression during the nematode life cycle. We then grouped these 6,695 probesets into 10 clusters based on their expression profiles (Figure 2). As an exemplary gene family, we analyzed the expression pattern of FMRF (Phe-Met-Arg-Phe-NH_2_)-related neuropeptide (FaRP)-encoding genes. This group encodes a specific class of neuronally expressed tetrapeptides that are potent myoactive transmitters in nematode neuromusculature [52-56], which are expressed in motor neurons that act on body wall muscle cells [57-59]. Based on our BLAST searches against various databases as detailed above, we identified five probesets for genes encoding FaRPs (HgAffx.23446.1.S1_at, HgAffx.23636.1.S1_at, HgAffx63.1.S1_at, HgAffx20469.1.S1_at, HgAffx.24161.1.S1_at). All five probesets were co-expressed with each other and showed an expression peak in the infective J2 stage (Additional data file 1). These FaRP probesets were differentially expressed when observed over the entire life cycle of the nematode, and, with the exception of HgAffx.20469.1.S1_at, which was found in cluster 4, all probesets were grouped in cluster 7 (Figure 2). The general profile of cluster 4 showed an expression peak in infective J2 and fell steadily in later life stages, while cluster 7 demonstrated the same overall pattern but showed a more pronounced increase from egg to infective J2.

Furthermore, we formed expression clusters for all 15 possible pairwise comparisons of all six life stages under study, as well as for comparisons of groups of life stages, that is: all pre-penetration (egg, infective J2) versus all post-penetration (parasitic J2, J3, J4, virgin females) life stages; and motile (pre-penetration J2) versus all non-motile parasitic (parasitic J2, J3, J4, virgin females) life stages. A summary displaying the number of probesets showing differential expression (FDR 5%) in these comparisons is given in Table 3.

**Table 3:** Differentially expressed probesets (FDR 5%)

Comparison	Number of probesets
Egg/infective J2	2,749
Infective J2/parasitic J2	3,012
Parasitic J2/J3	1,506
J3/J4	221
J4/female	1,136
Egg/female	4,588
Egg/parasitic J2	3,928
Egg/J3	4,415
Egg/J4	4,668
Parasitic J2/female	3,637
Parasitic J2/J4	2,320
J3/female	1,964
Infective J2/female	3,939
Infective J2/J3	3,489
Infective J2/J4	3,851
All pre-parasitic/all parasitic	5,178
All parasitic motile/all parasitic non-motile	4,137
All	6,695

**Figure 3:**
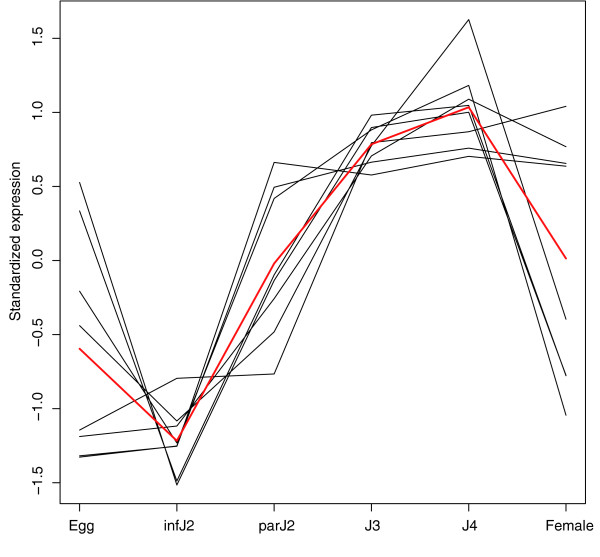
Temporal expression pattern of *H. glycines *probesets orthologous to *C. elegans *collagens. Reciprocal BLAST searches identified seven *H. glycines *probesets orthologous to *C. elegans *collagens. The average expression pattern of these seven probesets is indicated by a red line. For visualization purposes, each probeset's estimated mean log-scale expression profile was standardized to have mean 0 and variance 1.5 prior to plotting. infJ2, infective J2; parJ2, parasitic J2.

We used InterProScan [60] to conduct functional classification for all 6,860 contigs in all expression clusters of each comparison. The relative abundance of the 25 InterPro domains with the highest representation in each of the 10 clusters for contigs that showed differential expression (FDR 5%) throughout the entire life cycle is summarized in Additional data file 5. While most clusters contained a wide range of genes represented by diverse InterPro domains, collagen domains stood out, in that they accumulated in cluster 2 at a high frequency relative to other InterPro domains. Since it has been suggested that down-regulation of collagens might be a common feature in dauer and infective stages of nematodes [27], we analyzed the expression profiles of *H. glycines *collagens in more detail. Using a reciprocal BLAST strategy as described in Materials and methods, we identified eight *H. glycines *probesets representing seven unique contigs orthologous to *C. elegans *collagens (Table 4). The temporal expression pattern of these seven orthologs was very similar (Figure 3) and congruent with observations in other nematode species [14,51], which supports the hypothesis that down-regulation of collagen transcription is a conserved characteristic of non-molting infective and dauer-stage nematodes [27].

**Table 4:** *H. glycines *probesets orthologous to *C. elegans *collagens

*H. glycines *probeset	*C. elegans *collagen	E-value, score, % identity (BLASTX)**	E-value, score, % identity (TBLASTN)**
HgAffx.10090.1.S1_at*	CE06699	2e-25, 105, 59%	3e-24, 105, 59%
	CE05938	2e-25, 105, 59%	2e-25, 109, 57%
	CE05937	2e-25, 105, 59%	2e-25, 109, 57%
HgAffx.10017.1.S1_at	CE05147	1e-25, 106, 51%	2e-40, 159, 39%
HgAffx.18987.1.S1_at	CE32085	3e-32, 127, 66%	1e-30, 127, 66%
HgAffx.19573.1.S1_at	CE02380	4e-26, 108, 65%	4e-27, 115, 62%
HgAffx.19987.1.S1_at	CE02380	9e-30, 119, 62%	2e-28, 119, 62%
HgAffx.241.1.S1_at	CE29723	3e-32, 127, 56%	3e-32, 132, 52%
HgAffx.241.1.A1_at	CE29723	3e-32, 127, 58%	3e-32, 132, 52%
HgAffx.7962.1.S1_at*	CE04335	5e-82, 293, 69%	2e-87, 318, 85%
	CE04334	5e-82, 293, 69%	2e-87, 318, 85%

*Probeset matched several *C. elegans *collagens equally well.

**BLASTX of *H. glycines *nucleotide probesets against *C. elegans *collagen proteins and TBLASTN of *C. elegans *collagen proteins against *H. glycines *nucleotide probesets.

### *Heterodera glycines *orthologs of dauer-enriched *C. elegans *genes are more likely to be down-regulated upon transition from infective J2 to parasitic J2 and J3 than other genes

In addition to providing a comprehensive gene characterization and expression resource, we wished to demonstrate the applicability and power of our data by addressing the question of whether the infective J2 stage of *H. glycines *is biochemically analogous to the *C. elegans *dauer larva stage. We compiled a list of 1,839 *C. elegans *genes that were identified by Wang and Kim [61] as so-called dauer-regulated genes by conducting microarray experiments comparing gene expression in *C. elegans *larvae that were in transition from dauer to non-dauer with that of freshly fed L1 larvae that had been starved. Dauer-regulated genes showed significant expression changes during a dauer exit time course that were not related to the introduction of food [61]. Reciprocal BLAST searches resulted in the identification of 438 *H. glycines *probesets that could be categorized unambiguously as orthologs of these *C. elegans *dauer-regulated genes (Additional data file 6). Because of the deliberate redundancy of the Affymetrix GeneChip, these 438 probesets corresponded to 396 unique *H. glycines *gene predictions. In other words, we identified *H. glycines *orthologs for 22% of the 1,839 *C. elegans *dauer-regulated genes (396/1,839).

We also compiled a list of *H. glycines *genes that are orthologous to the 488 *C. elegans *gene subset of the *C. elegans *dauer-regulated genes that Wang and Kim [61] determined to be up-regulated during the dauer stage and down-regulated upon dauer exit, a group that was called dauer-enriched. These genes presumably define dauer-specific properties, including stress resistance and longevity. These dauer-enriched genes were of particular interest to us because up-regulation of orthologous genes in the *H. glycines *infective J2 stage would suggest involvement in developmental arrest of these genes not only in *C. elegans*, but also in *H. glycines*. Using the same reciprocal BLAST search strategy, we identified 74 *H. glycines *probesets corresponding to 69 unique *H. glycines *contigs or genes that are orthologous to 57 unique *C. elegans *dauer-enriched genes (Table 5), which represent 14%.

**Table 5:** *H. glycines *probesets orthologous to dauer-enriched *C. elegans *genes

*H. glycines *probeset	EST	Contig length	*C. elegans *gene	E-value, bit score, % identity (BLASTX*)	E-value, bit score, % identity (TBLASTN*)	Wormbase descriptor *C. elegans *gene	Cluster	InfJ2/parJ2^†^	InfJ2/J3^†^	J3/J4^†^
HgAffx.11262.2.S1_at	1	610	R151.2a	2e-53, 199, 73%	6e-52, 197, 79%	Phosphoribosyl pyrophosphate synthetase	1	Up	Up	
HgAffx.11331.1.A1_at	1	347	C25B8.3a	3e-26, 107, 85%	9e-25, 107, 85%	Peptidase C1A, papain	2	Down	Down	
HgAffx.11103.1.S1_at	3	745	C25B8.3a	4e-53, 184, 70%	4e-52, 184, 70%	Peptidase C1A, papain	2	Down	Down	
HgAffx.11103.1.A1_at	3	745	C25B8.3a	4e-53, 184, 70%	4e-52, 184, 70%	Peptidase C1A, papain	2			
HgAffx.11744.1.S1_at	4	656	Y17G7B.17	1e-19, 87, 32%	2e-17, 83, 35%	Proliferation-related protein MLF	1			
HgAffx.13580.1.S1_at	2	650	C10C6.5	1e-61, 226, 53%	1e-65, 244, 56%	ABC transporter	2			
HgAffx.15051.1.S1_at	16	807	F11G11.1	2e-41, 159, 42%	9e-41, 159, 42%	Collagen helix repeat	3		Down	
HgAffx.15051.2.S1_at	6	882	F11G11.2	2e-40, 157, 41%	2e-39, 155, 41%	Glutathione S-transferase	4		Down	
HgAffx.15228.1.S1_at	4	1,067	C46F4.2	e-143, 498, 67%	e-134, 473, 63%	AMP-dependent synthetase and ligase	5	Down	Down	
HgAffx.15789.1.S1_at	5	1,217	C46F4.2	5e-40, 155, 36%	6e-39, 155, 36%	AMP-dependent synthetase and ligase	2	Down	Down	
HgAffx.15789.2.S1_at	3	755	C46F4.2	2e-96, 342, 65%	3e-95, 342, 65%	AMP-dependent synthetase and ligase	2	Down	Down	
HgAffx.15812.1.S1_at	2	475	C51E3.6	1e-20, 90, 50%	7e-23, 102, 54%	Xanthine/uracil/vitamin C permease	2			
HgAffx.15725.1.S1_at	1	476	M110.5b	7e-56, 206, 63%	2e-51, 196, 62%	Pleckstrin homology-type	2	Down		
HgAffx.16156.1.S1_at	2	477	C53D6.7	7e-43, 163, 43%	4e-40, 158, 46%	Concanavalin A-like lectin/glucanase	2			
HgAffx.16267.1.S1_at	1	291	F11G11.2	3e-22, 94, 52%	6e-21, 94, 52%	Glutathione S-transferase	6	Up	Up	
HgAffx.17077.1.S1_at	8	1,012	B0361.9	5e-43, 165, 56%	9e-40, 156, 63%	N/apple PAN	3	Up	Up	
HgAffx.16890.1.S1_at	2	465	K07A3.2a	1e-23, 99, 47%	6e-22, 99, 47%	Sterol-sensing 5TM box	3	Up	Up	
HgAffx.16917.1.S1_at	2	616	F09G2.3	2e-29, 113, 54%	4e-28, 113, 54%	Phosphate transporter	5	Down	Down	
HgAffx.17264.1.S1_at	19	1,233	T03E6.7	7e-93, 331, 57%	3e-92, 331, 57%	Peptidase C1A, papain	3	Up	Up	
HgAffx.17605.1.S1_at	2	636	Y9C9A.16	6e-47, 177, 41%	7e-46, 177, 41%	FAD-dep. pyridine oxidoreductase	1		Down	
HgAffx.17530.1.S1_at	2	474	K08H10.4	6e-19, 84, 50%	1e-17, 84, 50%	Alpha-isopropylmalate synthase	2	Down		
HgAffx.17668.1.S1_at	1	481	K07C5.5	6e-32, 127, 44%	1e-30, 127, 44%	Epoxide hydrolase	2	Down	Down	
HgAffx.17855.1.S1_at	6	601	R13A5.3	6e-24, 101, 38%	2e-23, 101, 38%	Transthyretin-like	2			
HgAffx.18208.1.S1_at	7	803^‡^	K07C11.5	3e-21, 92, 32%	7e-20, 89, 33%	Netrin	5	Down	Down	
HgAffx.18170.1.S1_at	1	479	F39B3.2	2e-24, 102, 61%	9e-31, 127, 47%	Rhodopsin-like GPCR superfamily	5	Down	Down	
HgAffx.18607.1.S1_at	9	991	R11F4.1	e-126, 442, 67%	e-125, 442, 67%	Carbohydrate kinase	2		Up	Up
HgAffx.18847.1.S1_at	1	485	Y54G11A.5	8e-70, 251, 81%	2e-69, 251, 81%	Catalase	3		Up	
HgAffx.19435.1.S1_at	4	668	Y44F5A.1	2e-33, 133, 38%	1e-30, 127, 37%	WD-40 repeat	1	Down	Down	
HgAffx.19602.1.S1_at	1	340	C11E4.1	3e-34, 134, 67%	3e-33, 134, 67%	Glutathione peroxidase	7	Up	Up	
HgAffx.19847.1.S1_at	6	679	W01A11.6	5e-31, 125, 45%	2e-30, 125, 45%	Molybdenum biosynthesis protein	6	Up	Up	
HgAffx.19874.1.S1_at	1	484	R160.7	7e-36, 140, 58%	2e-34, 140, 58%	FYVE zinc finger	2	Down		
HgAffx.19903.1.S1_at	9	630	F45H10.4	4e-28, 115, 42%	2e-27, 155, 42%	Unnamed protein	2	Down		
HgAffx.20463.1.S1_at	1	395	F40E10.3	2e-40, 154, 55%	1e-59, 233, 76%	Calsequestrin	5	Down	Down	
HgAffx.20251.1.S1_at	1	395	C37C3.8b	7e-35, 136, 70%	5e-25, 108, 55%	Unnamed protein	6	Up		
HgAffx.20740.1.S1_at	4	574	T28B4.3	4e-31, 125, 50%	4e-22, 97, 40%	Transthyretin-like	5		Down	
HgAffx.20171.1.S1_at	2	885	T19B10.3	2e-58, 216, 39%	1e-58, 221, 40%	Glycoside hydrolase	2	Down		
HgAffx.20528.1.S1_at	2	653	K09C8.3	2e-28, 116, 33%	2e-19, 90, 31%	Peptidase M	3		Up	
HgAffx.20171.1.A1_at	2	885	T19B10.3	2e-58, 216, 39%	1e-58, 221, 40%	Glycoside hydrolase	2			
HgAffx.20464.1.S1_at	2	395	E02C12.4	2e-26, 108, 49%	6e-26, 109, 49%	Transthyretin-like	5	Down	Down	
HgAffx.20474.1.S1_at	5	695	C46F4.2	1e-23, 85, 55%	1e-17, 85, 55%	AMP-dependent synthetase and ligase	5			
HgAffx.20842.1.S1_at	15	1,556	C14F11.5	2e-54, 204, 67%	3e-76, 278, 62%	Heat shock protein Hsp20	3		Down	
HgAffx.21213.1.S1_at	7	1,329	C10C6.5	4e-32, 129, 36%	4e-31, 129, 36%	ABC transporter	7	Up		
HgAffx.22102.1.S1_at	2	556	CC4.2	3e-18, 82, 49%	3e-18, 84, 49%	Neuropeptide-like protein, nlp-15	5		Down	Down
HgAffx.22266.1.S1_at	3	435	C37H5.3a	3e-33, 131, 47%	9e-32, 131, 47%	Alpha/beta hydrolase	1	Down	Down	
HgAffx.22554.1.S1_at	5	1,194	H24K24.5	e-109, 387, 48%	e-101, 363, 45%	Dimethylaniline monooxygenase	3	Up	Up	
HgAffx.22270.1.S1_s_at	2	395	T05A7.1	2e-20, 89, 32%	2e-19, 89, 32%	Unnamed protein	5	Down	Down	
HgAffx.22723.1.S1_at	1	425	F21F3.1	2e-28, 115, 50%	5e-27, 115, 50%	Peptidyl-glycine monooxygenase	5	Down	Down	Down
HgAffx.22678.1.S1_at	11	707	K07E1.1	8e-61, 224, 61%	2e-59, 222, 63%	Acireductone dioxygenase	3	Up		
HgAffx.22868.1.S1_at	17	1,373	T03E6.7	e-120, 423, 62%	e-120, 423, 62%	Peptidase C1A, papain	2			
HgAffx.22801.1.S1_at	6	813	H10D18.2	1e-30, 124, 39%	1e-29, 122, 38%	Allergen V5/Tpx-1 related	5	Down	Down	
HgAffx.22840.1.S1_at	7	513	T10C6.14	8e-41, 157, 98%	8e-42, 161, 98%	Histone H4	5		Down	
HgAffx.22840.2.S1_at	6	501	T10C6.14	1e-41, 159, 97%	4e-41, 159, 97%	Histone H4	5		Down	
HgAffx.22798.1.S1_at	27	680	JC8.8	3e-43, 165, 58%	8e-43, 165, 58%	Transthyretin-like	1	Down	Down	
HgAffx.22879.1.S1_at	2	454	C11E4.2	7e-38, 85, 71%	7e-37, 85, 71%	Glutathione peroxidase	7	Up		
HgAffx.22709.1.S1_at	8	1,065	C28H8.6a	8e-85, 304, 58%	3e-84, 304, 58%	LIM, zinc-binding	5	Down	Down	
HgAffx.23565.1.S1_at	7	549	C11E4.1	1e-57, 213, 62%	1e-56, 212, 63%	Glutathione peroxidase	5	Down	Down	
HgAffx.23446.1.S1_at	5	876^‡^	F07D3.2	2e-19, 87, 51%	6e-18, 83, 38%	FMRFamide-related peptide	5		Down	Down
HgAffx.23377.1.S1_at	1	485	C33A12.7	7e-46, 173, 57%	7e-45, 173, 57%	Metallo-beta-lactamase superfamily	1	Down	Down	
HgAffx.24026.1.S1_at	5	541	T07C4.5	2e-23, 99, 46%	6e-23, 99, 46%	Transthyretin-like	5	Down	Down	
HgAffx.24221.1.S1_at	2	662	H14N18.3	3e-21, 92, 38%	3e-20, 90, 39%	Casein	1	Down	Down	
HgAffx.250.1.S1_at	7	908	F32A5.4a	3e-35, 139, 34%	4e-34, 137, 39%	Proteinase inhibitor I33, aspin	1	Down		
HgAffx.2812.1.S1_at	3	500	R11A8.4	2e-24, 102, 42%	2e-22, 100, 49%	Silent information regulator protein 2	2	Down	Down	
HgAffx.2812.1.A1_at	3	500	R11A8.4	2e-24, 102, 42%	2e-22, 100, 49%	Silent information regulator protein 2	1			
HgAffx.24343.1.S1_at	8	657	B0334.1	5e-28, 115, 46%	6e-31, 126, 49%	Transthyretin-like	7			
HgAffx.2920.1.S1_at	3	578	T10C6.14	3e-19, 85, 52%	1e-21, 94, 53%	Histone H4	3	Up	Up	
HgAffx.2920.1.A1_at	3	578	T10C6.14	3e-19, 85, 52%	1e-21, 94, 53%	Histone H4	3		Up	
HgAffx.2888.1.S1_at	2	473	T25B9.7	7e-25, 103, 35%	2e-23, 103, 35%	UDP-glucuronosyl/glucosyltransferase	3		Down	
HgAffx.2888.1.A1_at	2	473	T25B9.7	7e-25, 103, 35%	2e-23, 103, 35%	UDP-glucuronosyl/glucosyltransferase	4		Down	
HgAffx.4596.1.S1_at	1	573	C54D10.10	1e-18, 84, 43%	7e-18, 84, 43%	Proteinase inhibitor I2	3	Up	Up	
HgAffx.4485.1.S1_at	1	623	ZK945.1	5e-45, 171, 45%	8e-44, 171, 45%	Penicillin-binding protein	3	Up	Up	
HgAffx.5769.1.S1_at	1	620	K10B3.6a	2e-20, 89, 32%	5e-19, 89, 32%	Phosphodiesterase	3			
HgAffx.6540.1.S1_at	2	655	ZK945.1	4e-57, 211, 58%	5e-56, 211, 58%	Penicillin-binding protein	3	Up	Up	
HgAffx.8976.1.S1_at	2	694	T24D8.5	6e-18, 81, 70%	2e-17, 81, 70%	Neuropeptide-like protein, nlp-2	5	Down	Down	
HgAffx.9380.1.S1_at	2	756	Y54G11A.5	2e-84, 302, 60%	9e-86, 310, 62%	Catalase	1			

*BLASTX of *H. glycines *nucleotide probesets against *C. elegans *proteins and TBLASTN of *C. elegans *proteins against *H. glycines *nucleotide probesets. ^†^Differential expression (FDR 5%) where indicated and direction of expression change relative to infective J2. ^‡^Full-length sequence.

To test whether the frequency of *H. glycines *orthologs to *C. elegans *dauer-regulated and dauer-enriched genes is similar to that of other, randomly chosen genes, we randomly selected 1,000 *C. elegans *proteins from the Wormpep database (v. 157) and repeated the reciprocal BLAST searches. In these searches, we identified 159 unique *H. glycines *contigs that fulfilled our criteria (data not shown). In other words, 16% of these randomly selected *C. elegans *genes have *H. glycines *orthologs. These analyses showed that *C. elegans *dauer-regulated genes have a slightly higher frequency (22%) of having orthologs in *H. glycines *than either dauer-enriched (14%) or random (16%) genes, both having about the same rate.

**Figure 4:**
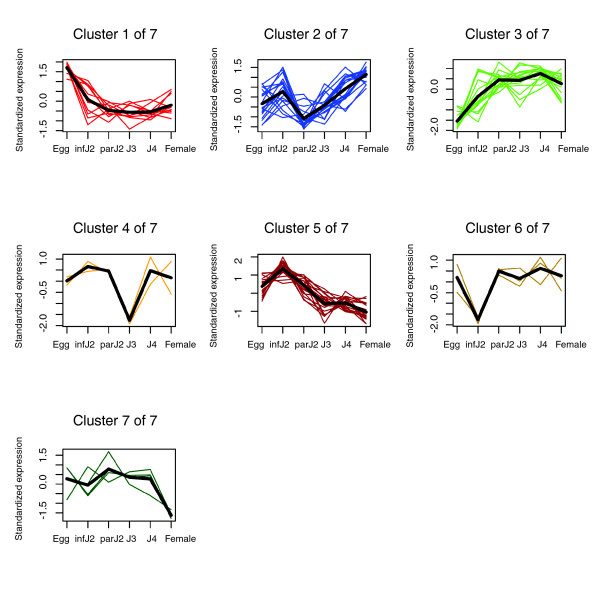
Temporal expression patterns of 74 *H. glycines *probesets orthologous to *C. elegans *dauer-enriched genes. Reciprocal BLAST searches identified 74 *H. glycines *probesets as orthologous to *C. elegans *dauer-enriched genes. These probesets were grouped into seven clusters based on their temporal expression profiles. The average expression pattern of the probesets in each cluster is indicated by a bold line. infJ2, infective J2; parJ2, parasitic J2.

Following the identification of *H. glycines *orthologs for *C. elegans *dauer-regulated and dauer-enriched genes, we clustered these genes according to their expression profiles throughout the life cycle. Clustering the 438 probesets for the dauer-regulated orthologs led to their placement into nine groups (Additional data file 2), while clustering of the 74 *H. glycines *probesets for dauer-enriched gene orthologs resulted in seven distinct groups (Figure 4). It is obvious that not all *H. glycines *dauer-enriched orthologs were down-regulated from infective J2 to parasitic J2. Indeed, only 41% out of 74 probesets were significantly down-regulated, whereas 22% were up-regulated and 38% did not exhibit a statistically significant change in expression. Similarly, when comparing the infective J2 stage with the J3 stage of *H. glycines*, only 47% out of 74 probesets were down-regulated. Twenty-two percent were up-regulated, and 31% did not exhibit a statistically significant change in expression. In other words, in both comparisons, the majority of *H. glycines *genes that are orthologous to *C. elegans *genes down-regulated upon dauer exit were up-regulated or did not exhibit a statistically significant change in expression.

To determine whether *H. glycines *genes orthologous to *C. elegans *dauer-enriched genes behave differently from other *H. glycines *genes, we compared the dauer-enriched *H. glycines *orthologs with the entire set of 7,530 *H. glycines *probesets on the Affymetrix Soybean Genome Array, as well as to the 159 *H. glycines *genes that we determined to be orthologous to 1,000 randomly chosen *C. elegans *genes. We found that out of 7,530 *H. glycines *probesets, 19% were down-regulated when infective J2 are compared to parasitic J2, 21% were up-regulated and 60% did not exhibit a statistically significant change. Similarly, when infective J2 are compared to J3, 26% of all probesets were down-regulated, 20% were up-regulated and 54% did not exhibit a statistically significant change. The 159 *H. glycines *genes that are orthologous to 1,000 randomly chosen *C. elegans *genes are represented by 181 probesets on the Affymetrix GeneChip. Of those 181, 27% were down-regulated from infective J2 to parasitic J2, 24% were up-regulated and 50% did not exhibit a statistically significant change. When infective J2 were compared to J3, 26% out of 181 were down-regulated, 20% up-regulated and 54% did not exhibit a statistically significant change. We used a Fisher's exact test [62] to examine whether the proportion of down-regulated genes among the set of dauer-enriched *H. glycines *orthologs was significantly different from all other genes on the array or from the *H. glycines *probesets that were orthologous to the 1,000 randomly chosen *C. elegans *genes, respectively. We found that the observed differences between the proportions of down-regulated probesets between dauer-enriched *H. glycines *orthologs and the entire set of probesets on the microarray were significant at the 0.05 level in comparisons of both infective J2 versus parasitic J2 (*P *= 0.000015) and infective J2 versus J3 (*P *= 0.007160). Similarly, the differences between dauer-enriched *H. glycines *orthologs and the *H. glycines *probesets orthologous to random *C. elegans *genes were significant for comparisons of infective J2 versus parasitic J2 (*P *= 0.0219190) and for infective J2 versus J3 (*P *= 0.0094261). If a Bonferroni correction is used to control the overall type I error rate for this family of four tests, all comparisons would remain significant at the 0.05 level except the comparison between dauer-enriched *H. glycines *orthologs and the *H. glycines *probesets orthologous to random *C. elegans *genes for infective J2 versus parasitic J2. In other words, while the majority of *H. glycines *genes that are orthologous to *C. elegans *dauer-enriched genes was not down-regulated upon transition to parasitic J2 or J3, the proportion of *H. glycines *orthologs that were in fact down-regulated was statistically significantly enriched about two times compared to all *H. glycines *genes on the microarray or to orthologs to random *C. elegans *genes.

The identities of *H. glycines *genes that followed the expression pattern of their dauer-enriched *C. elegans *orthologs (that is, they were down-regulated upon transition to infective J2 or J3) reflect a wide range of effector functions and biochemical pathways, including peptidases, epoxide and glycoside hydrolases, phosphate transporters and neuropeptide-like proteins. *H. glycines *genes that did not follow the *C. elegans *pattern of expression (that is, they were not down-regulated) span an equally diverse group of genes and include carbohydrate kinase, catalase and glutathione peroxidase (Table 5).

### Metabolism in *C. elegans *dauer larvae and *H. glycines *infective J2 is dissimilar

To investigate whether the infective J2 stage in *H. glycines *shows an expression profile of metabolic pathway genes similar to that of *C. elegans *dauer larvae, we conducted a BLAST search (threshold E = 1e^-20^) against the Wormpep database (v. 152) to search for Affymetrix probesets coding for *H. glycines *enzymes active in the citrate cycle, glycolysis and other pathways that undergo marked changes during the dauer state [31]. We identified 37 probesets coding for 24 proteins active in six different pathways (Table 6). We then compared the expression levels of these *H. glycines *probesets in the assayed *H. glycines *life stages and determined differential expression (FDR 5%). While phosphofructokinase has been found to be up-regulated in dauer larvae relative to adults in *C. elegans *[40], we could not find differential expression between infective J2 and adult females in *H. glycines*. The citrate cycle is down-regulated in the *C. elegans *dauer stage and active at a lower level than the glyoxylate pathway [40,41]. In *H. glycines*, out of eight genes for citrate cycle enzymes found, all but one (fumarase) showed differential expression in at least one out of three stage-by-stage comparisons (egg/infective J2, infective J2/feeding J2, infective J2/female). However, while pyruvate dehydrogenase and nucleoside diphosphate kinase were down-regulated in infective J2 (which supports similar metabolic patterns in *H. glycines *J2 and *C. elegans *dauer larvae), isocitrate dehydrogenase, citrate synthase, succinyl-CoA synthetase and succinate dehydrogenase were up-regulated in this stage when compared to the other life stages tested (which points to significant differences between *H. glycines *and *C. elegans*). The gene encoding malate dehydrogenase was up-regulated in infective J2, which is concordinant with observations of high malate dehydrogenase enzyme activity in *C. elegans *dauer larvae relative to adults. Of genes encoding three enzymes of the glyoxylate pathway, two (citrate synthase and malate dehydrogenase) were differentially expressed between infective J2 and eggs, feeding J2 or adult females. Both enzymes are shared with the citrate cycle. Even though both citrate synthase and malate dehydrogenase transcripts were up-regulated in infective J2, their expression level did not support observations of a higher activity of the glyoxylate pathway, as described for *C. elegans *dauer larvae [41] in infective J2 when compared to other citrate cycle enzymes. Apart from these pathways, the dauer stage in *C. elegans *is known to have high expression levels of DAF-21/Hsp90, superoxide dismutase and catalase to counter environmental stressors [31]. In *H. glycines*, none of these dauer metabolism hallmarks could be confirmed. The gene encoding Hsp90 was differentially expressed in the infective J2 versus parasitic J2 comparison and was down-regulated in infective J2. The superoxide dismutase gene was differentially expressed and down-regulated in infective J2 when compared to eggs, parasitic J2 and adult females. Catalase-2 was differentially expressed when compared to eggs (up-regulated in infective J2) and females (down-regulated in infective J2), and catalase-3 was differentially expressed and down-regulated in infective J2 when compared to eggs. In summary, we conclude from these data that the physiological and biochemical landscape of developmentally arrested *C. elegans *dauer larvae must be different from that of developmentally arrested *H. glycines *infective J2.

**Table 6:** *H. glycines *probesets homologous to *C. elegans *genes that are involved in dauer metabolism

*H. glycines *probeset	EST	Contig length	E-value*	Identity (%)	*C. elegans *gene	Descriptor *C. elegans *gene	InfJ2/egg^†^	InfJ2/parJ2^†^	InfJ2/female^†^
**Phosphofructokinase**									
HgAffx.14924.1.S1_at	6	670	2.4e-77	72.1	Y110A7A.6a	Phosphofructokinase	Up	Up	
									
**Citrate cycle**									
HgAffx.18932.1.S1_at	7	882	1.4e-113	76.8	T05H10.6a	Pyruvate dehydrogenase E1 alpha subunit		Down	
HgAffx.22652.1.S1_at	16	488	5.8e-73	83.9	T20G5.2	Citrate synthase	Up		
HgAffx.17812.1.S1_at	4	493	8.1e-56	76.1	T20G5.2	Citrate synthase	Up		
HgAffx.6732.1.S1_at	1	631	3.9e-74	71.3	C37E2.1	Isocitrate dehydrogenase	Down	Up	Up
HgAffx.22639.1.S1_at	6	489	6.6e-45	62.1	C34F6.8	Isocitrate and isopropylmalate dehydrogenases	Up	Up	Up
HgAffx.15376.1.S1_at	1	435	3.8e-38	75.2	C30F12.7	Isocitrate dehydrogenase			Up
HgAffx.13764.1.S1_at	1	400	9.2e-30	60.7	C05G5.4	Succinyl-CoA synthetase	Up	Up	Up
HgAffx.23207.1.A1_at	4	604	3.9e-31	46.4	F25H2.5	Nucleoside diphosphate kinase	Up	Down	Down
HgAffx.24228.1.S1_at	17	790	1.2e-59	75.4	F25H2.5	Nucleoside diphosphate kinase		Down	
HgAffx.14699.1.S1_at	4	666	2.5e-82	71	C03G5.1	Succinate dehydrogenase subunit			Up
HgAffx.23633.1.S1_at	3	624	8.8e-75	77.6	F42A8.2	Succinate dehydrogenase	Up		Up
HgAffx.18264.1.S1_at	1	472	5.2e-50	76.4	C03G5.1	Succinate dehydrogenase subunit			Up
HgAffx.23633.2.S1_at	1	406	1.7e-33	82	F42A8.2	Succinate dehydrogenase	Up		Up
HgAffx.17082.1.S1_at	1	375	4.4e-31	51.1	T07C4.7	Succinate dehydrogenase cytochrome b chain		Up	Up
HgAffx.2164.1.A1_at	2	738	8.4e-82	76	H14A12.2a	Fumarase			
HgAffx.2164.1.S1_at	2	738	8.4e-82	76	H14A12.2a	Fumarase			
HgAffx.17587.1.S1_at	1	488	4.2e-45	62.7	F20H11.3	Malate dehydrogenase	Up		Up
HgAffx.14431.1.S1_at	3	551	1.0e-57	67.6	F20H11.3	Malate dehydrogenase	Up		
									
**Glyoxylate cycle**									
HgAffx.22652.1.S1_at	16	488	5.8e-73	83.9	T20G5.2	Citrate synthase	Up		
HgAffx.17812.1.S1_at	4	493	8.1e-56	76.1	T20G5.2	Citrate synthase	Up		
HgAffx.21758.1.S1_at	2	402	2.9e-37	77.7	C05E4.9a	Isocitrate lyase			
HgAffx.17587.1.S1_at	1	488	4.2e-45	62.7	F20H11.3	Malate dehydrogenase	Up		Up
HgAffx.14431.1.S1_at	3	551	1.0e-57	67.6	F20H11.3	Malate dehydrogenase	Up		
									
**SMA and DAF proteins**									
HgAffx.22024.1.S1_at	5	772	1.6e-78	74.2	ZK370.2	SMA-2			
HgAffx.17831.1.S1_at	1	423	1.1e-41	67.0	R13F6.9	SMA-3			Down
HgAffx.19781.1.S1_at	1	463	3.7e-49	69.9	R12B2.1	SMA-4	Down	Up	Up
HgAffx.16166.1.S1_at	1	475	3.9e-26	50.0	C32D5.2	SMA-6			Down
HgAffx.10752.1.S1_at	1	651	9.5e-30	44.7	B0240.3	DAF-11			Up
HgAffx.8385.1.S1_at	1	410	4.5e-21	77.5	R13H8.1a	DAF-16			
HgAffx.21332.1.S1_at	36	2325	9.0e-280	78.6	C47E8.5	DAF-21 (Heatshock protein 90)		Down	
									
**Superoxide dismutase**									
HgAffx.23145.1.S1_at	9	691	1.1e-51	60.7	C15F1.7a	Superoxide dismutase 1		Down	
HgAffx.18049.1.S1_at	3	698	2.2e-38	60.9	ZK430.3	Superoxide dismutase 5	Down	Down	Down
HgAffx.7684.1.S1_at	2	538	1.7e-55	67.3	F10D11.1	Superoxide dismutase 2	Down		
HgAffx.18572.1.S1_at	2	522	6.5e-24	41.6	ZK430.3	Superoxide dismutase 5			Down
									
**Catalase**									
HgAffx.18847.1.S1_at	1	485	6.4e-67	81.9	Y54G11A.5	Catalase 2	Up		Down
HgAffx.9380.1.S1_at	2	756	1.2e-57	64.6	Y54G11A.13	Catalase 3	Down		

*Threshold 1e-20. ^†^Differential expression (FDR 5%) where indicated and expression level of probeset in infective J2 relative to other stage.

### Validation of microarray results by quantitative RT-PCR

Quantitative real-time reverse transcription PCR (qRT-PCR) was used to validate selected microarray results. We analyzed the expression patterns of six genes representing different expression patterns for each of the five consecutive pairs of life stages (egg/infective J2, infective J2/parasitic J2, parasitic J2/J3, J3/J4, J4/female), giving a total of 30 different genes. The qRT-PCR template was the same biological material used for hybridization of the microarrays, and the reactions were performed in technical triplicates. Additional data file 7 shows that there is qualitative agreement between our microarray approach and qRT-PCR for 28 out of 30 genes.

## Discussion

This study describes the generation of the most exhaustive EST collection of the soybean cyst nematode *H. glycines *to date and the analyses and characterization of this EST collection. The Affymetrix Soybean Genome Array GeneChip contains a significant portion of probesets for *H. glycines *genes and became a possibility only because of the extensive EST collection generated in this project. This GeneChip now is the first commercially available microarray for a nematode other than *C. elegans*. We analyzed the expression profiles of all 6,860 genes throughout all major developmental stages, excluding the adult male. Furthermore, we classified all genes by predicted function and conducted stage-wise comparisons to identify differentially expressed genes. Finally, these data now represent a resource for any molecular project targeting *H. glycines*, and we have demonstrated the versatility of this genomics resource by advancing our understanding of arrested development in the infective stage.

It has been proposed that the *C. elegans *genome can serve as a guide to examine aspects of the biology of other nematode species, particularly those that are parasitic [26], and we have shown that this comparative genomics approach has great power. In particular, we examined whether the biochemistry underpinning the developmentally arrested, infective J2 stage of *H. glycines *is functionally analogous to that of the dauer stage in *C. elegans*. For this purpose, we exploited published microarray expression data obtained from *C. elegans *during dauer exit [61]. These down-regulated genes, termed 'dauer-enriched,' exhibit high mRNA abundance during the dauer stage. We asked if these genes were conserved in *H. glycines*, both in sequence and expression pattern. While our reciprocal BLAST searches suggest that a portion of *C. elegans *dauer-enriched genes is indeed conserved in the *H. glycines *genome with about the same frequency as randomly selected *C. elegans *genes, we did not find that *H. glycines *orthologs of *C. elegans *dauer-enriched genes are uniformly down-regulated upon transition to the parasitic J2 or J3 stages. While in *C. elegans *dauer-enriched genes are down-regulated upon dauer exit [61], we found that only 41% of their *H. glycines *orthologs are down-regulated upon transition to the feeding J2 stage, which we hypothesize is a developmental transition equivalent to dauer exit in *C. elegans*. Nevertheless, *H. glycines *dauer-enriched orthologs are more likely to be down-regulated than: all genes represented on the microarray; and *H. glycines *orthologs for randomly selected *C. elegans *genes. In other words, while our data do not support the idea of a broadly conserved gene expression signature between the dauer stage in *C. elegans *and infective J2 in *H. glycines*, they indicate that dauer-enriched orthologs are more likely to share a common expression profile than other genes. A similar preliminary observation was made in an EST study [27] that compared the infective stage of the human-parasitic nematode *S. stercoralis *and dauer-specific transcripts that were identified in serial analysis of gene expression (SAGE) in *C. elegans *[51]. Mitreva *et al*. [27] found that dauer-specific genes were conserved in *S. stercoralis*, but that there was no evidence of a broadly conserved expression signature. However, in this study, we were able to compare our exhaustive microarray data for *H. glycines *with microarray data for *C. elegans*, which enables us to draw more compelling conclusions regarding dauer-regulated genes in both species.

We further compared the expression of metabolic pathway genes of *C. elegans *dauer larvae with infective J2 of *H. glycines*. Our data for *H. glycines *genes whose products are active in the glyoxylate pathway or citrate cycle, both of which undergo marked gene expression changes in *C. elegans *dauer larvae, as well as for genes encoding Hsp90 or superoxide dismutase, show dramatic differences between *H. glycines *infective J2 and *C. elegans *dauer larvae. Our findings suggest that the *C. elegans *dauer larva and the *H. glycines *infective J2 do not share similar expression profiles of metabolic pathway genes.

Although based upon inferences from transcript levels, our data point to striking differences in the underlying biochemistry and physiology of developmentally arrested and recovering *C. elegans *dauers and *H. glycines *J2. Does the phenomenon of developmental arrest in these stages therefore reflect a substantially different regulatory pathway, or does it reflect life-style differences between the parasitic and free-living species? Until the genome of *H. glycines *is complete, it will not be possible to determine if more putative orthologs of the *daf *and *sma *genes that compose the *C. elegans *dauer pathway are present than we were able to identify here. Many of these regulators of dauer entry/exit [31] are typically expressed at low levels, and thus are not common in EST sets.

Numerous nematode species are carried passively to new habitats as eggs and achieve active dispersion as dauer larvae or infective stage. This active dispersal stage in many nematodes is thought to have evolved from a common ancestral stage, and it has been assumed that there has been sufficient conservation of gene expression over time that the expression patterns in different species would appear similar. Our findings of marked differences in gene expression between *C. elegans *dauer larvae and *H. glycines *infective J2 could mean that these two developmentally arrested stages have evolved independently from each other and that all apparent similarities are based on convergent evolution. Alternatively, and we believe more likely, is that these two stages could in fact have a common origin but molecular evolution could have been sufficiently fast that any broadly conserved gene expression patterns would have been lost since the last common ancestor. Perhaps the best example of rapid diversification of genetic and biochemical processes underlying an analogous biological process comes from comparisons of vulval induction in *C. elegans *and *Pristionchus pacificus *[63]. In both species, vulva induction occurs post-embryonically via an identical cellular process (inductive signaling to P5.p, P6.p and P7.p cells from the anchor cell). Despite the obvious homology of these processes, the underlying regulation is strikingly distinct [63]. Similarly, other studies have demonstrated that gene expression patterns between mouse and human, which split only about 25 million years ago, changed rapidly [64-66].

Concerning the *C. elegans *dauer stage and potentially analogous stages in parasitic nematodes, it will be interesting to define the mechanisms that lead to developmental arrest in *H. glycines *and to compare them further to processes in *C. elegans*. The comprehensive dataset generated here will be a valuable resource for the field of nematology and sets the stage for many more comparative studies. In particular, a comprehensive analysis of *H. glycines *parasitism-associated gene expression profiles has been conducted by these authors and will be published later.

## Conclusion

Our data indicate that expectations of a conserved phylum-wide dauer expression signature shared across nematodes may not be realistic. Such general expectations severely underestimated the degree to which expression profiles change and should be replaced by careful analyses of dauer-like stages of closely related nematode species instead. For example, one could ask whether the dauer expression profiles of *C. elegans *and *Caenorhabditis briggsae *are the same or whether the expression profiles of *H. glycines *infective J2 and *M. incognita *infective J2 are conserved.

## Materials and methods

### Nematode cultivation, cDNA library generation, sequencing and clustering

*H. glycines *strain OP-50 [67] was cultivated under greenhouse conditions and isolated as described previously [68]. Unidirectional Uni-Zap lambda libraries (Stratagene, La Jolla, CA, USA) were generated for *H. glycines *strain OP-50 eggs, infective J2, J3, J4 and virgin females. The mRNA concentration used ranged between 1.0 μg (J3) and 4.6 μg (eggs). The libraries were sequenced at the Washington University Medical School Genome Sequencing Center (St Louis, MO, USA), and the sequencing results were deposited in GenBank. Contigs were formed by Affymetrix for the design of the *H. glycines *group of probesets of the Affymetrix Soybean Genome Array GeneChip and all consensus sequences and contig size details can be accessed at Affymetrix [69].

### Nematode cultivation for microarray experiments

For each replication, 40 pots with 10 seeds each of Kenwood 94 soybeans were planted in a 2:1 sand:soil mixture in the greenhouse. Two weeks after planting, each pot, containing an average of 7 to 8 germinated seedlings, was inoculated with 15,000 to 20,000 *H. glycines *strain OP-50 [67] infective J2. The inoculum was collected by setting up two hatch chambers, each containing about two million *H. glycines *OP-50 eggs, and allowing the eggs to hatch for four days. From the same batch of eggs used in the hatch chamber, 50,000 eggs were collected and flash frozen in liquid nitrogen for use as the egg stage of the replication. After 4 days, the hatched infective J2 were collected and counted, and an aliquot of 50,000 larvae was flash frozen in liquid nitrogen for use as the infective J2 stage of the replication. The remainder of hatched infective J2 larvae was divided up among the 40 pots for seedling inoculation. Four days after infection, 12 pots were collected, and the soil was washed away from the root systems of these pots to isolate the parasitic J2 stage. Eight days after infection, another 12 pots were harvested for collection of J3 juveniles, and, 14 days after infection, a further 10 pots were used to isolate J4 juveniles. Finally, 21 days after infection, the final 6 pots were harvested for collection of adult females. These stages were isolated as published previously [68]. All stages were divided in about 30 mg aliquots in 1.5 ml screw cap tubes, flash frozen in liquid nitrogen and stored at -80°C.

### RNA extraction for microarray experiments and GeneChip procedures

Frozen (-20°C) 1.0 mm zirconia beads (BioSpec Products, Bartlesville, OK, USA) were added to frozen nematode tissue in a 1.5 ml screw cap tube in a 1:1 tissue:bead ratio. RNA was isolated using the Versagene kit from Gentra Systems (Minneapolis, MN, USA). On ice, 800 μl lysis buffer was mixed with 8 μl tri 2-carboxyethyl phosphine (TCEP), and 50 μl was added to one screw cap tube with about 30 mg nematode tissue. Tissue was homogenized in a beadbeater (BioSpec Products) at setting 48 for 10 s and chilled on ice for 60 s. This step was repeated three times. Two more tubes for the same life stage were treated in the same way. The suspension was collected by pipetting and transferred into a new tube. Beads were rinsed with remaining buffer, and the standard Versagene protocol was followed from then on. For all stages and all repetitions, we obtained 113 to 320 mg frozen tissue and 7.85 to 173.38 μg total RNA. The RNA concentration and quality of each sample were determined by a NanoDrop spectrophotometer (NanoDrop Technologies, Wilmington, DE, USA) and by RNA Nanochip on a 2100 Bioanalyzer (Agilent Technologies Inc., Palo Alto, CA, USA). RNA was submitted to the Iowa State University GeneChip Facility, where standard procedures recommended by Affymetrix were followed for reverse transcription and labeling of the probes and for hybridization and scanning of the GeneChips.

### Experimental design of microarray experiments and GeneChip data analysis

Expression was measured using a total of 18 Affymetrix Soybean Genome Array GeneChips (three replications × six life stages) using a randomized complete block design with replications as blocks. Prior to performing the analysis, the Affymetrix signal data were transformed using the natural log (ln) and normalized by median centering (that is, the median ln signal from each particular chip was subtracted from all ln signals on the chip). The normalized data for each gene were analyzed separately using a standard linear model with fixed effects for replications and stages. F tests, resulting in *p-*values, were performed to test for a difference in expression between the life stages for each probeset. A q-value was computed for each *p-*value using the method described by Storey and Tibshirani [70]. These q-values can be used to identify differential expression while maintaining approximate control of the FDR. For example, FDR is controlled at approximately 5% if the sets of tests with q-values at or below 0.05 are declared significant. See [71] for a discussion of linear modeling and FDR control in the context of plant microarray experimentation.

Clustering was used to organize and summarize the observed expression patterns of differentially expressed genes. For each probeset, the mean normalized expression level was estimated for all six life stages. The six estimated values were standardized to have mean 0 and standard deviation 1 within each probeset. The Euclidian distance between any pair of standardized expression profiles was used as a measure of dissimilarity in all clustering algorithms. This approach considers genes with similar expression patterns to be close in six-dimensional space and is equivalent to using (1-r)^0.5 ^as the measure of dissimilarity, where r is the Pearson correlation coefficient between non-standardized expression profiles.

K-medoids clustering [72] was performed on those probesets with the 1,000 lowest *p*-values (< 0.0000143) for the overall test for expression change across the life cycle. Using the Gap statistic [73], we determined the number of clusters to be 10. Probesets with q-values less than 0.05 were added to these 10 base clusters to produce the clustering depicted in Figure 2. All other clusters were produced using hierarchical agglomerative clustering, using average linkage to measure the dissimilarity between clusters.

All clusters and related figures were generated using the free open-source statistical software package R [74]. Hierarchical clustering was carried out using the R function hclust; K-medoids clustering was carried out using the function pam from the R cluster library.

### BLAST searches of *H. glycines *contigs

We built three nematode-specific nucleotide databases as follows: cyst nematodes (13,643 sequences) - all sequences from the GenBank query "*Globodera *[ORGN] or *Heterodera *[ORGN] not *Heterodera glycines *[ORGN]"; non-cyst nematodes (933,882 sequences) - results of the GenBank query "Nematoda [ORGN] not *Globodera *[ORGN] not *Heterodera *[ORGN]"; and non-nematodes (3,607,410 sequences) - GenBank 'nt' database (nucleotide version of 'nr') minus all Nematoda [ORGN] sequences. The BLASTN parameters were set to expect a 75% target frequency as follows: M = 1, N = -1, Q = 3, R = 3, B = 10, V = 10, lcmask, golmax = 10, topcomboN = 1, filter = seg. The following parameters were used for BLASTX searches of all 6,860 contigs against non-redundant GenBank (downloaded 29 November 2005) and Wormpep v. 152: filter = seg, lcfilter, W = 4, T = 20, E = 100, B = 25, V = 25, topcomboN = 1, golmax = 10.

### InterProScan

InterProScan was run using InterPro data files [75] dated November 2005 (iprscan_PTHR_DATA_12.0.tar). InterProScan translated all 6,860 unique contig sequences in six frames and then ran its suite of domain-finding tools. We required a minimum translation length of 20 amino acids to be considered by InterProScan, and we used the EGC.0 translation table. Due to the six-frame translation, each contig typically had several alignments amongst the significant open reading frame (ORF) found in the translation. We kept, as representative of each contig, the single longest aligning ORF that contained an InterPro domain, even though InterProScan may have found several ORFs for each contig with alignments to some domain or motif. Results were parsed into files representing expression clusters.

### Identification of *C. elegans *dauer and collagen orthologs

A previous microarray study identified 1,984 so-called dauer-regulated and 540 dauer-enriched genes in *C. elegans *[61]. We manually compiled a list of 1,839 dauer-regulated and 488 dauer-enriched *C. elegans *genes using these data (the remaining genes were either deleted or merged with other genes in Wormbase). To isolate *H. glycines *orthologs, we conducted BLAST searches between these *C. elegans *genes and all 7,530 *H. glycines *probeset nucleotide sequences (threshold values 1^e-15^, bit score at least 50, identity at least 30%). We then conducted an additional BLAST search between the *H. glycines *probesets that passed those criteria and the entire Wormpep (v. 159) database using the same cutoff criteria as above. Only those ortholog pairs in which the *C. elegans *dauer-regulated or dauer-enriched hit was either the best one compared to *C. elegans *hits of the entire Wormpep database or within 5% of the best hit's bit score were kept. These searches generated a list of 438 *H. glycines *probesets orthologous to *C. elegans *dauer-regulated genes and 74 *H. glycines *probesets orthologous to *C. elegans *dauer-enriched genes.

To identify collagen orthologs, we downloaded the sequences for collagens listed at the Sanger Institute web site [76] and followed basically the same BLAST strategy, with the exception that we required a higher stringency with a bit score of at least 100.

### qRT-PCR

The transcript abundance of 30 differentially expressed cDNA clones was analyzed by qRT-PCR to confirm microarray results. Gene-specific primers were designed, and the sequences are shown in Additional data file 7. DNase-treated RNA (10 ng) was used for cDNA synthesis and PCR amplification using an iScript One-Step RT-PCR kit (BIO-RAD, Hercules, CA, USA) according to manufacturer's protocol. The PCR reactions were performed using an iCycler (BIO-RAD) under the following conditions: 50°C for 10 minutes, 95°C for 5 minutes and 40 cycles of 95°C for 30 s and 60°C for 30 s. Following PCR amplification, the reactions were subjected to temperature ramp to create the dissociation curve, measured as changes in fluorescence as a function of temperature, by which the non-specific products can be detected. The dissociation program was 95°C for 1 minute, 55°C for 10 s, followed by a slow ramp from 55°C to 95°C. Three replicates of each reaction were performed, and constitutively expressed *Actin 1 *(AF318603) was used as internal control to normalize gene expression levels. Quantifying the relative changes in gene expression was performed using the 2^-ΔΔ*CT *^method as described by Livak and Schmittgen [77].

### Data

The Affymetrix Soybean Genome Array GeneChip raw and normalized data files were deposited in the ArrayExpress database [78] under accession number E-MEXP-1110. This database is MIAME-compliant.

GenBank accession numbers for EST generated in this study: BF013452-BF014867, BF249436-BF249525, BG310659-BG310919, BI396450-BI397002, BI451481-BI451765, BI704104-BI704170, BI749028-BI749665, BI773548-BI773559, CA939105-CA940989, CB238504-CB238733, CB238735-CB238737, CB238740, CB238743-CB238746, CB238750-CB238751, CB238755-CB238759, CB238763, CB238766-CB238769, CB238772-CB238774, CB238776-CB238778, CB238780-CB238782, CB238784, CB238788, CB238790-CB238793, CB278389-CB280465, CB281104-CB281884, CB299031-CB299936, CB373779-CB376363, CB377726-CB380382, CB824093-CB826589, CB934836-CB935636, CD747803-CD749135.

## Abbreviations

EST, expressed sequence tag; FaRP, FMRF (Phe-Met-Arg-Phe-NH_2_)-related neuropeptide; FDR, false discovery rate; Hsp, heatshock protein; J, juvenile stage; ORF, open reading frame; qRT-PCR, Quantitative real-time PCR.

## Authors' contributions

AAE planned and coordinated the study, prepared tissue for microarrays, analyzed the data and wrote the manuscript. MM and JPMcC oversaw sequencing and sequence analyses, analyzed data and edited the manuscript. JR assisted in statistical analyses. XG and JM assisted in sequence analyses. TRM generated tissue samples. JPMcD constructed cDNA libraries. TH performed qRT-PCR. DMB analyzed data and edited the manuscript. ELD provided material. RSH edited the manuscript. DN performed and directed statistical analyses and edited the manuscript. TJB planned the study, analyzed data and wrote the manuscript.

## Additional data files

The following additional data are available with the online version of this paper. Additional data file 1 is a figure showing the temporal expression pattern of *H. glycines *probesets for FaRP-encoding genes. Additional data file 2 is a figure showing temporal expression patterns and clusters of 438 *H. glycines *probesets orthologous to *C. elegans *dauer-regulated genes. Additional data file 3 is a table listing the 25 most conserved *H. glycines *contigs compared to *C. elegans*. Additional data file 4 is a table of identities and cluster membership of differentially expressed (FDR 5%) probesets over the entire life cycle. Additional data file 5 is a table listing the 25 most abundant InterPro domains for the 10 expression clusters for *H. glycines *genes that are differentially expressed over the entire life cycle. Additional data file 6 is a table listing *H. glycines *probesets orthologous to dauer-regulated *C. elegans *genes and their cluster memberships. Additional data file 7 is a table listing qRT-PCR results, oligonucleotide primers used and probeset identities. Additional data file 8 provides transformed and normalized Affymetrix probeset mean data for each probeset in each life stage and q values for each tested combination of life stages as described in Materials and methods.

## Supplementary Material

Additional data file 1 BLAST searches identified seven *H. glycines *probesets orthologous to *C. elegans *FaRP-encoding genes (mean expression pattern indicated in red). For visualization purposes, each probeset's estimated mean log-scale expression profile was standardized to have mean 0 and variance 1.5 prior to plotting. infJ2, infective J2; parJ2, parasitic J2. Click here for file

Additional data file 2 Reciprocal BLAST searches identified 438 *H. glycines *probesets as orthologous to *C. elegans *dauer-regulated genes. These probesets were grouped into nine clusters based on their temporal expression profiles. The average expression pattern of the probesets in each cluster is indicated by a bold line. infJ2, infective J2; parJ2, parasitic J2. Click here for file

Additional data file 3 The 25 most conserved *H. glycines *contigs compared to *C. elegans*. Click here for file

Additional data file 4 Identities and cluster membership of differentially expressed (FDR 5%) probesets over the entire life cycle. Click here for file

Additional data file 5 The 25 most abundant InterPro domains for the 10 expression clusters for *H. glycines *genes that are differentially expressed over the entire life cycle. Click here for file

Additional data file 6 *H. glycines *probesets orthologous to dauer-regulated *C. elegans *genes and their cluster memberships. Click here for file

Additional data file 7 qRT-PCR results, oligonucleotide primers used and probeset identities. Click here for file

Additional data file 8 Transformed and normalized Affymetrix probeset mean data for each probeset in each life stage and q values for each tested combination of life stages as described in Materials and methods. Click here for file
